# Overview on the Role of Advance Genomics in Conservation Biology of Endangered Species

**DOI:** 10.1155/2016/3460416

**Published:** 2016-11-29

**Authors:** Suliman Khan, Ghulam Nabi, Muhammad Wajid Ullah, Muhammad Yousaf, Sehrish Manan, Rabeea Siddique, Hongwei Hou

**Affiliations:** ^1^The Key Laboratory of Aquatic Biodiversity and Conservation, Chinese Academy of Sciences, Institute of Hydrobiology, Chinese Academy of Sciences, Wuhan, Hubei 430072, China; ^2^Department of Biomedical Engineering, Huazhong University of Science and Technology, Wuhan 430074, China; ^3^Center for Human Genome Research, Cardio-X Institute, Huazhong University of Science and Technology, Wuhan 430074, China; ^4^National Key Laboratory of Crop Genetic Improvement, College of Plant Sciences and Technology, Huazhong Agricultural University, Wuhan 430070, China; ^5^Institute of Biotechnology and Genetic Engineering, The University of Agriculture, Peshawar 25000, Pakistan

## Abstract

In the recent era, due to tremendous advancement in industrialization, pollution and other anthropogenic activities have created a serious scenario for biota survival. It has been reported that present biota is entering a “sixth” mass extinction, because of chronic exposure to anthropogenic activities. Various* ex situ* and* in situ* measures have been adopted for conservation of threatened and endangered plants and animal species; however, these have been limited due to various discrepancies associated with them. Current advancement in molecular technologies, especially, genomics, is playing a very crucial role in biodiversity conservation. Advance genomics helps in identifying the segments of genome responsible for adaptation. It can also improve our understanding about microevolution through a better understanding of selection, mutation, assertive matting, and recombination. Advance genomics helps in identifying genes that are essential for fitness and ultimately for developing modern and fast monitoring tools for endangered biodiversity. This review article focuses on the applications of advanced genomics mainly demographic, adaptive genetic variations, inbreeding, hybridization and introgression, and disease susceptibilities, in the conservation of threatened biota. In short, it provides the fundamentals for novice readers and advancement in genomics for the experts working for the conservation of endangered plant and animal species.

## 1. Introduction

Anthropogenic activities have changed the global environment, reducing the biodiversity through extinction and also reducing the population size of already surviving species. Due to man-made activities and interruptions, the current rate of species extinction is 1,000 times higher than natural background rates of extinction and future rates are likely to be 10,000 times higher [1].* According to IUCN 2015 report*,* currently 79*,*837 species were assessed*,* of which* 23,250 are threatened with extinction. Only one-third of the world's freshwater fishes are at risk from hydropower dam expansion [2]. According to various estimates, each year few thousands to 100,000 species extinct, most without ever having been scientifically described [3]. Due to these tremendous anthropogenic activities, the notion has been emerged that earth biota is entering a “sixth” mass extinction [4] which is based on the facts that recent rates of species extinction are very high than prehuman background rates [5, 6].* Only in the Island of Tropical Oceana*,* 1800 bird species were reported to extinct* in approximately 2000 years, since human colonization [7]. Even in the scientifically advanced* 19th and 20th centuries*,* numerous species of birds*,* mammals*,* reptiles*,* fresh water fishes*,* amphibians*,* and other organisms extinction have been documented* [5, 8, 9].* If species extinction persists at such a tremendous speed*, future generation will occupy a planet with significantly reduce biodiversity, diminished ecosystem services, reduced evolutionary potential, and ultimately higher extinction rate and collapse ecosystem [3, 10].

It is a major challenge for biologists and ecologists to protect endangered species. Several measures have been taken and efforts done in this regard which is extensively described in literature such as population viability analysis, formulation of metapopulation theory, species conservation, contribution of molecular biology, development of global position system, geographical information system, and remote sensing [11].* In the recent era*,* genomics is a key part of all the biological sciences and* a quickly changing approach to conservation biology. The genomes of many thousands of organisms including plants, vertebrates, and invertebrates have been sequenced and the results augmented, are annotated, and are refined through the use of new approaches in metabolomics, proteomics, and transcriptomics that enhance the characterization of metabolites, messenger RNA, and protein [12]. The genomic approaches* can provide detail information about the* present and past demographic parameters, phylogenetic issues, the molecular basis for inbreeding, understanding genetic diseases, and detecting hybridization/introgression in organisms [13]. It can also provide information to understand the mechanisms that relate low fitness to low genetic variation, for integrating genetic and environmental methodologies to conservation biology and for designing latest, fast monitoring tools. The rapid financial and technical progress in genomics currently makes conservation genomics feasible and will improve the feasibility in the very near future even [14]. The objective of this review is to describe recently advanced molecular technologies and their role in species conservation. We have described the effectiveness and possibility of conservation technology using the advance genomic approaches along with their limitations and future development. We hope that this review will provide fundamentals and new insights to both new readers and experienced biologists and ecologists in formulating new tools and establishing technologies to prevent endangered species.

## 2. Biodiversity and Conservation 

Biodiversity refers to the variety of all forms of life on this planet, including various microorganisms, plants, animals, the ecosystem they form, and the genes they contain. Biodiversity within an area, biome, or planet is therefore considered at three levels including species diversity, genetic diversity, and ecosystem diversity [15]. As the names indicate, species diversity refers to the variety of species; genetic diversity is the variation of genes within species and populations and ecosystem diversity relates to the variety of habitats, ecological processes, and biotic communities in the biosphere [15]. Today's biodiversity about 9.0 to 52 million species is the result of billions of years of evolution, shaped by natural phenomena, and forms the web of life of which we are an integral part and upon which we are so fully [15, 16]. For species adaptation and survival, genetic diversity is the basic element and all the evolutionary achievement and to some degree survival depend on it. Though both adaptation and survival can be viewed in terms of space, time, and fitness but fitness further includes adaptation, genetic variability, and stability. The phenomenon of extinction can be the result of either abiotic or biotic stresses, caused by various factors such as disease, parasitism, predation, and competition or due to habitat alteration or isolation due to human activities, natural catastrophes, and slow climatic and geological changes. Considering these persistent threats, it is very crucial that genetic diversity in species should be appropriately understood and efficiently conserved and used [17].

At present, several species are in retreat, losing localities, and increasingly threatened with extinction by various factors mainly human intervention, and thus conservation biology has become a major file in recent times. A “threatened” designation generally recognizes a significant risk of becoming endangered throughout all or a portion of a species' range. Although extinction is a natural process, the human understanding of the value of the endangered species and its realization to intervene the stability of the environment is rapidly increasing. Human interferes in the natural environment of species in different ways, such as destruction of natural habitat, the introduction of nonnative organisms, and direct killing of natural components of a population [18]. Maintaining natural variation of species is beneficial from an economical, ecological, and social perspective. Several combinations of benefit occur for any particular species, and some species are obviously more valuable than the others.

Currently, the maintenance of rare and endangered species is a main focus of interest of biologists and geneticists. The impact of extinction is not always apparent and difficult to predict, and thus several parameters have been set and different technologies are being developed. For example, population viability analysis (PVA) quantitatively predicts the probability of extinction and prioritizes the conservation needs. It takes into account the combined impact of both stochastic (including the demography, environment, and genetics) and terministic (including habitat loss and overexploitation) factors [11]. Mandujano and Escobedo-Morales using PVA method for howler monkeys (*Alouatta palliata mexicana*) to simulate a group trend and local extinction and to investigate the role of demographic parameters to population growth under two landscape scenarios isolated populations and metapopulation [19]. They found that the rate of relative reproductive success and fecundity is directly linked with the number of adult females per fragment. As a result, the finite growth rate depended mainly on the survival of adult females while in both isolated populations and metapopulation the probability of extinction was exponentially dependent on fragment size. Further, it establishes a minimum viable population, predicts population dynamics, establishes conservation management programs, and evaluates its strategies. However, it is limited by several factors; for example, it is often very difficult to measure small-population parameters which need to be used in PVA models. This necessitates the development of more comprehensive and well-established approaches that can not only predict the extinction but also predict rather at a very early stage.

## 3. Role of Genomics Analysis Tools in Species Conservation

The term genome is about 75 years old and refers to the total set of genes on chromosomes or refers to the organism complete genetic material [20]. Together with the effect of an environment, it forms the phenotype of an individual. Thomas Roderick in 1986 coined the term genomics as a scientific discipline which refers to the mapping, sequencing, and analysis of the genome [21]. Now due to universal acceptance of genomics, it expands and is generally divided into functional and structural genomics. Structural genomics refers to the evolution, structure, and organization of the genome while functional genomics deals with the expression and function of the genome. Functional genomics needs assistance from structural genomics, mathematics, computer sciences, computational biology, and all areas of biology [22].

**Figure 1:**
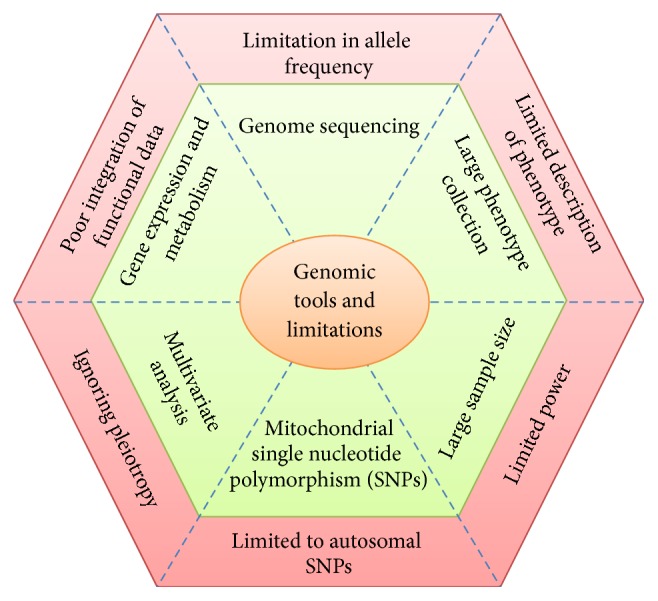
Illustration of various genetic tools for detection of genetic variations in species and their limitations in broad spectrum applications.

**Figure 2:**
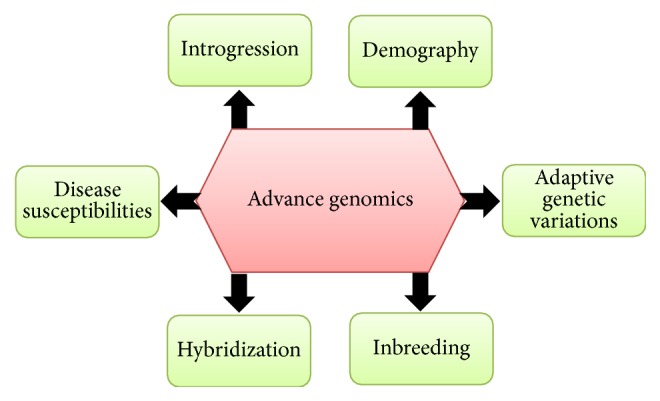
Illustration of advance genomic approaches for the conservation of species.

Genome analysis was once limited to model organisms [23] but now the genomes of thousands of organisms including plants, invertebrates, and vertebrates have been sequenced and the results annotated are further refined and augmented by using new approaches in metabolomics, proteomics, and transcriptomics [12]. Nowadays, it is quite easier to investigate the population structure, genetic variations, and recent demographic events in threatened species, using population genomic approaches. With recent developments, hints for becoming endangered species can be found in their genome sequences. For example, any deleterious mutations in the genes for brain function, metabolism, immunity, and so forth can be easily detected by advanced genomic approaches. Conversely, these can also detect any changes in their genome which may result in enhanced functions of some genes, for example, related to enhanced brain function and metabolism that may lead to the abnormal accumulation of toxins [24–26]. Specific genetic tools and analytical techniques are used to assess the genome of various species to detect genetic variations associated with specific conservation and population structure. Currently, most commonly used genetic tools for detection of genetic variations in both plant and animal species include random fragment length polymorphism (RFLP), amplified fragment length polymorphism (AFLP), random amplification of polymorphic DNA (RAPD), single strand conformation polymorphism (SSCP), minisatellites, microsatellites, single nucleotide polymorphisms (SNPs), DNA and RNA sequence analysis, and DNA finger printing. Analysis of genetic variation in species or population using these tools is carried out either using current DNA of individuals or historic DNA [27]. These tools target different variables within the genome of target species and selection of the specific tools and gnome part to be analyzed is carried out based on the available information. For example, mitochondrial DNA in animals possessing a high substitution rate is a useful marker for the determination of genetic variations in individuals of the same species. However, these techniques have several limitations associated with them. For instance, genetic high substitution rate in animal mitochondrial DNA is only inherited in female lines. Similarly, the mitochondrial DNA in plants has a very high rate of structural mutations and thus can rarely be used as genetic marker for detection of genetic variation. Various genomic tools used for the detection of genetic variations in species and limitations associated with them are summarized in Figure 1. Genome-wide association studies (GWAS), development of genome-wide genetic markers for DNA profiling and marker assisted breeding, and quantitative trait loci (QTL) analysis in endangered and threatened species can give us information about the role of natural selection at the genome level and identification of loci linked with the disease susceptibility, inbreeding depression, and local adaptations. For example, most of the QTLs have been detected using linkage mapping and cover large segments of the genome in different species. Currently, due to the availability of high-density SNP chips and genome-wide analysis techniques, GWAS has proven to be effective in identification of important genomic regions more precisely within the genome of species, for example, those associated with genetic variations and important qualitative and quantitative traits [28]. Further, use of population genetics and phylogenomics can help us in identifying conservation units for recovery, management, and protections [23]. As the genome of more species is sequenced, the rescue of more endangered species will become easier. The applications of advance genomics in the conservation of threatened biota are illustrated in Figure 2.

### 3.1. Demography

To identify recent and historic demographic events such as geographic population structure, gene flow, admixture, and population size fluctuations, specific genetic markers such as silent sites and microsatellites have been traditionally used. Although traditional molecular approaches have successfully analyzed and modeled complex demography histories, effective population size, nucleotide diversity, and recombination, genomics have provided a greater statistical and analytical power [29]. Genomics can also provide information about speciation time, recombination rates, origin, relationship, and estimation of current and ancestral effective population size [30, 31]. Similarly, population genomics can improve our understanding about microevolution through a better understanding of recombination, assertive matting, mutation, and selection which helps us in identifying genes that are crucial for adaptation and fitness [32]. Future genetic analysis using SNPs can be of more advantage in determination of genome structure in regions with high linkage disequilibrium (LD) and low haplotype number in order to accelerate and optimize gene mapping based on genetic association, for example, finding relatively frequent variants associated with complex traits. However, this requires extensive knowledge of the LD patterns in the genome. It has been suggested that LD in genomes can be organized as a pattern of blocks of different length possessing limited diversity and separated by regions of low LD. Such structure can be the result of a number of possible mechanisms, one of which is recombination hotspots [33].

### 3.2. Adaptive Genetic Variations

Selective forces shape adaptive variations and identification of these adaptive loci is one of the most crucial focuses of genomics in conservation and evolution [34]. Genomics can help us to identify genetic changes resulting from local adaptation and the way these alterations influence fitness, through access to genome-wide data and annotated genomes in wild species. This information will not only help in defining conservation units [35] but also provide information about population potentials to respond to changing environmental condition [36]. Similarly, understanding the relationship between local adaptation and geographic distribution of loci will also benefit to evaluate habitat requirements for population persistence and the ecological exchangeability of divergent populations [37].

Various techniques are used to identify genetic regions associated with the adaptive traits. The most frequently used method is QTL [38], which has been used for many wild species such as cave tetra fish [39], deer mouse [40], and the zebra finch [41]. For example, the yield improvement of several crops such as wheat and maize has been made possible through the indirect manipulation of QTLs that control the heritable variability of the traits and physiological mechanisms [42, 43]. The conventional approaches of crop improvement such as breeding were based on little or no knowledge of the factors governing the genetic variability [44]. However, the conventional approaches for determining the genetic diversity are currently insufficient as the factors, for example, abiotic factors, including heat, stress, drought, water logging, and salinity, are becoming more prevalent in certain areas. Consequently, the genetic dissection of quantitative traits controlling the adaptive response in important crops to abiotic stresses is essential to allow cost-effective applications of genomic-based approaches to breeding programs aimed at improving the sustainability and stability of yield under adverse conditions [43]. Due to limited life history availability, nowadays, RAD-sequence [13, 45], GWAS [46, 47], and genome scan [48–50] are also used for identification of genetic regions associated with the adaptive traits. For example, GWAS was applied across the whole genome in several crops to detect the nonrandom association between the genomic markers scattered across the genome and the adaptive trait of interest [51]. Historical recombination increases the resolution in the detection of the locus controlling the adaptive traits [52], and thus GWAS identifies the nonrandom association of alleles among a locus with the adaptive traits (i.e., LD) as a result of action of natural selection [53]. The Major Histocompatibility Complex (MHC) has a role in kin recognition, intraspecific territoriality, and mate choice [54] and identification of polymorphism in MHC loci through genomics can give us information about the immunological fitness of the population [55] and further as advances are made can help us in conservation managements.

### 3.3. Inbreeding

 Inbreeding of a species results in inbreeding depression, which can cause reduction of evolutionary adaptive potential, and ultimately increases the risk of extinction [56] but the exact mechanism of how this leads to the inbreeding depression is poorly understood. Only it is the genomics, which can shed light on the genetic architecture of inbreeding depression, a number of loci that contribute to inbreeding, and some underlying genetic mechanisms such as epistasis, overdominance, dominance, and/or gene-environment interactions [23]. For example, small scabious, a perennial plant in Netherlands, is an endangered species with highly fragmented and genetically eroded populations. Various transcriptomics and epigenetic analyses of inbreeding and inbreeding depression have been used to analyze this plant in the context of conservation. Various methods such as GWAS [57], gene expression profiles [58], and sequencing the whole genome of both parents and offspring [59] are used to identify loci related to inbreeding depression. The most immediate effect of inbreeding in a population is to reduce the frequency of heterozygotes, lowered fitness of individuals, inbreeding depression [60], and loss of diversity due to genetic drift by reducing effective population size [61]. For example, Mooney and McGraw studied* Panax quinquefolius* (American ginseng), a rare plant for outcrossing and inbreeding [62]. For inbreeding, the* Panax quinquefolius were either self-pollinated or* either cross-pollinated within the population. On the other hand for outcrossing,* Panax quinquefolius were* either cross-pollinated within the population or with cultivated plants. Offspring resulted from all the crosses were followed for 4 years. Seedlings from self-pollinated plants showed 33% smaller heights and 45% smaller leaf areas relative to those from cross-pollination. On the other hand, Seedlings from crosses with cultivated plants showed 165% greater root biomass and 127% greater leaf area relative to outcrosses within the population. This example shows how inbreeding accelerates population extinction.

### 3.4. Hybridization and Introgression

Hybridization in some plant taxonomic group requires molecular markers at the genome level due to the peculiar characteristics of their genome architecture. For example, the wild form of sunflower, a noxious weed, can serve as a weed to the crop form. Hybridization can take place through pollinating insects which can move some of the crop's pollens into the weed populations. Ongoing hybridization between closely related species appears to be common in nature [63]. Genomics can provide better insight in the roles of hybridization and introgressive gene flow in natural populations and also can clear our concept of how species can maintain their genetic distinctiveness and reproductive isolation. Because introgressive gene flow may decrease or increase fitness, a better capability to identify the timing and occurrence of gene flow between species is relevant to population management and sustainability [23]. Translocated populations sometimes can hybridize with closely related or native populations of the same species, compromising the genetic purity of each species. For example, when* Cervus nippon* (Sika deer) were introduced to Western Europe, they readily inbreed with native* C. elaphus* (red deer) and as a result in Great Britain, there are no pure red deer [64]. Some extent of genetic flow is through a normal and evolutionarily constructive process, as the entire constellations of genotypes and genes cannot be preserved. However, hybridization with or without introgression in a rare or threatened species may compromise their existence. In this regard, only advance molecular technologies can play a significant role to understand the underappreciated problem that is not always evident from morphological observations alone [65].

### 3.5. Disease Susceptibilities

Infectious diseases, especially viral ones, are generally considered as a cause of decline in population [66] and are seldom considered a cause of extinction. In conservation biology, except in unusual circumstances, infectious diseases have a contributory or marginal influence on extinction [67, 68]. Recently it has been found that long term exposure to infectious diseases may alter the constitution of genome [69] which has a role in evolution and shaping of our biochemical individuality [70]. Advanced genomics can identify relevant susceptible genes and can provide better comprehensions into protective and pathogenic mechanisms and can pinpoint new molecular targets for therapeutic and prophylactic interventions [71]. Genome-wide SNP studies and whole genome sequencing can provide better understanding in wild life species managements and treatment of diseases [72] as the immediate goal for conservation management is to assess carrier status and to provide the basis for species recovery [73]. Currently, there are various examples under threat for various reasons being severely impacted by infectious diseases such as canine distemper in lions and black-footed ferrets [74, 75], Marburg and Ebola hemorrhagic diseases in anthropoids [76], transmissible facial tumor disease in Tasmanian devils [77], Kola retrovirus [78], and* Chlamydia pecorum* in Koalas [79]. In conservation biology, though host-pathogen interaction is a subject of particular interest, the possibility that pathogen causes extinction in certain context is rarely understood. However, increasing developments in the molecular technologies can provide substantial contribution to precisely understand the microbiological processes in wildlife [80].

## 4. Future Hope from Advance Genomics

Approximately, one-quarter of all avian species are either nearly threatened or threatened. Only 73 species of which are rescued from extinction. One of them was* Nipponia nippon* (crested ibis), which only from seven individuals was recovered, using high-quality genome sequences [25]. Even scientists for the first time created a viable clone of the world smallest endangered sheep,* European mouflon*, providing a hope to save them from extinction. Similarly, the original gene pool of any extinct population can be regenerated* via* cloning, by preserving their genetic diversity, through collection of cell samples. Even if cloning is managed properly, it may expand the genetic pool, can help us bring back genetic materials from dead animals, infertile animals, and even young animals that were too immature to breed [81]. For those extinct organisms for which no living cell exists, cloning is impossible; however, genome editing is the only means to bring extinct species or more accurately extinct traits back to life [82].

## 5. Limitations of Genomics

The most important impediment in conservation genomics is either lack of availability of samples or difficulty in sample collections of endangered species. Similarly, production of genomics data is easier and faster, but data analysis technique mostly lags. In addition, many statistical programs for population genetics need to be adapted to large data sets and require significant advances in bioinformatics and computational biology. Application of genetic data may result in defining units of conservation too narrowly, may impede conservation actions, and may stand in the way of endangered species management [83]. Further, genomes of some endangered species have not been sequenced yet and this requires not only heavy funds but considerable time. Some policies relating to sample exchange among countries also retard the speed of biological conservation.

## 6. Conclusions and Future Prospects

Conservation genetics is mainly focused on to determine the relationship between species or population, study the cross-species variation, and describe the interactions between species and their threatening processes. In the current manuscript, we have overviewed the problems of conservation of endangered species and possible solutions and genetic and genomic approaches to apprehend them. Besides preventing the threatened species, diversity can benefit from looking beyond these and considering the genome of rare species and others that share a common environment. By identifying the factors or processes that influence the genomic composition of the threatened or extinction species, we can predict and identify the ecologically and genetically unique species. In the future, we hope that as advancement continues in genomics, we will be able to accurately predict the viability of local population and also to predict the ability of populations to adapt to climatic change and other anthropogenic challenges. Both climatic changes and anthropogenic activities due to a population explosion will be increasing day by day. Therefore, both these factors are a serious menace to biodiversity loss. The only hope to prevent their loss is expected from advance genomics. Further studies are also needed to appropriately understand and utilize environmental and genomic data and better ways to integrate them with multidisciplines, including policy analysis for effective conservation. Further, special policies should be established, to exchange the samples and genetic data of endangered species, in order to enhance species survival by the efforts of multinational groups.
